# Hyperbaric oxygen activates visfatin expression and angiogenesis via angiotensin II and JNK pathway in hypoxic human coronary artery endothelial cells

**DOI:** 10.1111/jcmm.14926

**Published:** 2020-01-19

**Authors:** Chiung‐Zuan Chiu, Bao‐Wei Wang, Ying‐Ju Yu, Kou‐Gi Shyu

**Affiliations:** ^1^ School of Medicine College of Medicine Fu Jen Catholic University New Taipei Taiwan; ^2^ Division of Cardiology Shin‐Kong Wu Ho‐Su Memorial Hospital Taipei Taiwan; ^3^ Central Laboratory Shin‐Kong Wu Ho‐Su Memorial Hospital Taipei Taiwan

**Keywords:** adipocytokine, angiogenesis, hyperbaric oxygen, hypoxia

## Abstract

Visfatin is an adipocytokine with important roles in endothelial angiogenesis. Hyperbaric oxygen (HBO) has been widely used to treat various medical illness with enhanced angiogenesis. The molecular effects of HBO on visfatin under hypoxia are poorly understood. This study aimed to investigate the effect of HBO on visfatin in hypoxic human coronary arterial endothelial cells (HCAECs). HCAECs under chemical hypoxia (antimycin A, 0.01 mmol/L) were exposed to HBO (2.5 atmosphere absolute; ATA) for 2‐4 hours. Western blot, real‐time polymerase chain reaction, electrophoretic mobility shift assay, luciferase promoter activity, migration and tube formation assay, and in vitro glucose uptake were measured. Visfatin protein expression increased in hypoxic HCAECs with earlier angiotensin II (AngII) secretion and c‐Jun N‐terminal kinase (JNK) phosphorylation, which could be effectively suppressed by the JNK inhibitor (SP600125), AngII antibody or AngII receptor blocker (losartan). In hypoxic HCAECs, HBO further induced earlier expression of visfatin and AngII. Hypoxia significantly increased DNA‐protein binding activity of hypoxia‐inducible factor‐1α (HIF‐1α) and visfatin. Hypoxia, hypoxia with HBO and exogenous addition of AngII also increased promoter transcription to visfatin; SP600125 and losartan blocked this activity. In HCAECs, glucose uptake, migration and tube formation were increased in the presence of hypoxia with HBO, but were inhibited by visfatin small interfering RNA, SP600125 and losartan. In conclusion, HBO activates visfatin expression and angiogenesis in hypoxic HCAECs, an effect mediated by AngII, mainly through the JNK pathway.

## 1 INTRODUCTION

Visceral fat accumulation has been shown to play important roles in the development of cardiovascular diseases.^1^, ^2^ Fat tissue is an endocrine organ producing adipocytokines that influence energy and lipid metabolism, insulin sensitivity, blood pressure, inflammation and angiogenesis.^1^ The well‐known adipocytokine, visfatin, was initially demonstrated to improve insulin sensitivity.^3^ Further researches^4^, ^5^ showed that visfatin expression increased by hypoxia, enhancing angiogenesis in adipocytes and breast cancer cells through hypoxia‐inducible factor‐1α (HIF‐1α). Visfatin was also shown to be associated with the expression of fibroblast growth factor 2 (FEF‐2), signal transducer and activator of transcription 3 (STAT3), vascular endothelial growth factor (VEGF), matrix metalloproteinase (MMP) and monocyte‐chemoattractant protein‐1 (MCP‐1), all of which enhance angiogenesis.^6^, ^7^, ^8^, ^9^, ^10^ Angiogenesis is activated by visfatin in endothelial cells (ECs) through HIF‐1α by increased cell migration and tube formation.^6^, ^7^, ^8^, ^10^, ^11^, ^12^, ^13^ Under hypoxia, ECs stimulate angiogenesis by expression of cardiac‐restricted genes through various signalling cascades.^14^, ^15^, ^16^, ^17^, ^18^ Models in vascular smooth muscle cells (VSMCs) and cardiomyocytes revealed that visfatin reduced cell death following exposure to hypoxia.^19^, ^20^ Hypoxia also stimulates pro‐inflammatory cytokines, such as angiotensin II (AngII), to mediate visfatin‐induced angiogenesis.^4^, ^5^ AngII has been shown to induce visfatin expression in ECs and cardiomyocytes.^21^, ^22^

However, the effect of hypoxia on visfatin‐mediated angiogenesis is not yet fully understood in human coronary artery ECs (HCAECs). Hyperbaric oxygen (HBO) therapy provides an increase in oxygen supply in hypo‐perfused tissues^23^ and enhances wound healing by increasing fibroblastic replication, collagen synthesis and neovascularization..^24^, ^25^, ^26^ However, the effect of HBO on visfatin in hypoxic HCAECs to enhance angiogenesis has not been explored. Our previous study showed that HBO‐induced visfatin expression in HCAECs is mediated by tumour necrosis factor‐*α* (TNF‐*α*) through the c‐Jun N‐terminal kinase (JNK) pathway.^27^ Thus, visfatin and the JNK pathway may be key mediators of angiogenesis in HCAECs under HBO. The aim of this study was to investigate the molecular mechanisms mediating visfatin expression by HBO in hypoxic HCAECs. We have been suggested that HIF‐1α may play a role in the transcriptional regulation of visfatin in hypoxic HCAECs treated with HBO.

## 2 MATERIAL AND METHODS

### 2.1 Primary HCAEC culture

HCAECs were purchased from PromoCell GmbH company and were then cultured in EC growth medium supplemented with foetal bovine serum (FBS, 10%), penicillin (100 U/mL) and streptomycin (100 µg/mL) at 37°C in a humidified atmosphere with 5% CO_2_. HCAECs were grown up to 90% in confluency in 10‐cm^2^ culture dishes and were then sub‐cultured at a ratio of 1:2.

### 2.2 Chemical hypoxia

Chemical hypoxia was generated by using 0.01 mmol/L antimycin A from Sigma‐Aldrich with glucose‐free medium (Dulbecco's Modified Eagle Medium; DMEM) as described previously.^28^, ^29^, ^30^ HCAECs were then rinsed with serum‐free DMEM, pyruvate, glutamine, non‐essential amino acids and glucose, containing NaHCO_3_ (3.7 g/L) and HEPES (20 mmol/L) at pH 7.4 after confluency. HCAECs were then incubated in the medium either with or without antimycin A for 0.5‐4 hours. In previous studies, the optimal exposure time and the suitable concentration of antimycm A were suggested.^28^, ^29^, ^30^ For our experiments, cells were pre‐incubated with inhibitors and reagents for 30 minutes, washed and exposed to antimycin A for different hour.

### 2.3 HBO treatment

The oxygen tension and atmosphere absolute (ATA) for HBO were used based on previous treatment protocol^26^ and our previous study.^27^, ^31^ HCAECs were exposed to 2.5 ATA of oxygen (98% O_2_ and 2% CO_2_) in a temperature‐controlled (37°C) hyperbaric chamber for 2‐4 hours. (Figure S1).

### 2.4 Antibodies, inhibitors and reagents

Different signal pathways, including JNK, p38 mitogen‐activated protein kinase (MAPK), extracellular‐signal‐regulated kinase (ERK) and phosphatidylinositol‐3 kinase (PI‐3K), in regulating hypoxia‐related visfatin expression were detected by pre‐treatment of HCAECs with SP600125 (20 mmol/L; a JNK pathway inhibitor), SB203580 (3 μmol/L; a p38 MAPK inhibitor), PD98059 (50 μmol/L; an ERK inhibitor) or wortmannin (50 nmol/L; a PI‐3K inhibitor) 30 min, respectively, before hypoxia. All of above inhibitors were purchased from CALBIOCHEM^®^. Polyclonal antibodies against visfatin were purchased from AdipoGen. Mouse monoclonal antibody against phospho‐JNK was purchased from Santa Cruz Biotechnology. Rabbit polyclonal antibodies against JNK were purchased from Cell Signaling Technology. AngII was obtained from Bachem AG. AngII type 1 receptor blocker (ARB; 100 nmol/L) was obtained from Merck & Co., Inc. N‐Acetyl cysteine (NAC; a reactive oxygen species [ROS] inhibitor) was purchased from CALBIOCHEM^®^. Interleukin‐6 (IL‐6) and TNF‐*α* were purchased from PeproTech. L‐NAME (L‐arginine methyl ester; an inhibitor of nitric oxide [NO] synthase) was purchased from Merck Millpore. The working concentration of NAC, IL‐6, TNF‐*α* and L‐NAME was 1 mmol/L, 10 μg/mL, 300 pg/mL and 300 μmol/L, respectively.

### 2.5 Alternative method for total RNA extraction from HCAECs

Total RNA was extracted from HCAECs by using a TRI reagent. Total RNA was extracted from HCAECs using Spin Columns system by a total RNA purification kit (cat. No.217004, Qiagen) following the manufacturers' protocols. The kit is designed to facilitate lysis of tissues, to inhibit RNases and also to remove most of the cellular DNA and proteins from the lysate. Further, the total RNA quantification was assessed by measuring the ratio of spectrophotometric absorbance (260 nm/280 nm). For a pure RNA sample, this ratio should be comprised between 1.8 and 2.

### 2.6 Reverse transcription quantitative PCR

Reverse transcription quantitative PCR (RT‐qPCR) was performed by using a Lightcycler purchased from Roche Diagnostics. Two genes (visfatin as study group and alpha‐Tubulin as control group) were used in this study. The primer sequences of visfatin are forward: 5′CCACCgACTCgTACAAg3′ and reverse: 5′gTgAgCCAgTAgCACTC3′. The primer sequences of alpha‐Tubulin are forward: 5′gATCACCAATgCTTgCTTTgAg3′ and reverse: 5′ACCATggCgAggg‐ TCACAT 3′. We used delta Ct (cycle threshold values) method to calculate the expression ratio in PCR. The primer efficiencies were evaluated by performing a 10‐fold dilution series experiment using the target assay. After properly setting the baseline and threshold, the slope of the standard curve can be translated into primer efficiency value through ABI Real‐Time PCR System version 2.0 software programs. Primers' specificity has been identified by derivative reporter (‐Rn) through melting curve analysis. Total 1 µg RNA was incubated with Moloney‐murine leukaemia virus (M‐MuLV) reverse transcriptase (Finnzyme; 200 U) in a buffer containing 50 mmol/L Tris‐Cl with PH 8.3, KCl (75 mmol/L), MgCl2 (3 mmol/L), RNase inhibitor (20 U), poly‐dT oligomer (1 µmol/L) and dNTP (0.5 mmol/L) in a total volume of 20 µL. The reaction was incubated at 42°C for 1 hour and followed by at 94°C for 5 minutes. Diethyl pyrocarbonate‐treated water (80 µL) was added to the reaction mixture before storage at −70°C. 1 μg of RNA was reverse‐transcribed by the M‐MuLV reverse transcriptase in a total volume of 20 μL. The reverse‐transcribed product was amplified with the DyNAmo HS SYBR Green qPCR Kit (Finnzyme) in the reaction mixture containing DyNAmo SYBR Green master mix and primers. Diluted cDNA (1 in 10) and a Lightcycler SYBR Green mastermix solution containing 0.5 μmol/L primer, 5 mmol/L MgCl_2_ and 2 µL Master SYBR Green in nuclease‐free water (Roche Diagnostics) were used for RT‐qPCR. The denaturation phase was 5 minutes at 95°C. The amplification phase was as below: denaturation at 95°C for 10 seconds; annealing at 63°C for 7 seconds; elongation at 72°C for 8 seconds; and detection at 79°C and for 45 cycles. Amplification plots, fluorescence detection and numbers of technical replicates and cycles were finally detected by using the Lightcycler apparatus.

### 2.7 Western blot analysis

HCAECs were homogenized in a lysis buffer (Promega Corp.) and were then centrifuged at 10 600 *g* for 20 minutes at 4°C. The protein content of the supernatant was measured by using the Bio‐Rad Protein Assay with BSA as the standard. The lysate was then incubated with a polyclonal anti‐visfatin antibodies for 2 hours at 4°C, followed by precipitation on protein A–agarose beads. The immunoprecipitated proteins were washed three times with lysis buffer before SDS/PAGE. Western blot analysis was performed in brief as following. Equal amounts of protein (15 μg) were mixed with sample buffer, boiled for 10 minutes, separated by SDS/PAGE under denaturing conditions and electroblotted on to nitrocellulose membranes. The blots were incubated overnight in TBS (Tris‐buffered saline) containing skimmed milk (5%) to block non‐specific binding of the antibodies. Proteins of interest were revealed with specific antibodies at 1:1000 dilutions for 1 hour at 22°C, followed by incubation with HRP (horseradish peroxidase)‐conjugated polyclonal anti‐rabbit IgG antibodies (1:5000 dilution) for 1 hour at room temperature. Antibody binding was detected by using ECL^®^ (Amersham Biosciences). Equal protein loading of the samples was verified by staining with a mouse anti‐tubulin monoclonal antibody (Sigma‐Aldrich Technologies). All Western blots were finally quantified by densitometry.

### 2.8 RNA interference

HCAECs were transfected with JNK‐ or visfatin‐annealed small interfering RNA (siRNA; Dharmacon). JNK and visfatin siRNAs are target‐specific siRNAs designed to suppress gene expression. The sense and antisense siRNA sequences of JNK were 5′‐CGUGGAUUUAUGGUCUGUGdTdT and 5′‐ CACAGACCAUAAAUCCACGdTdT, respectively. The sense and antisense siRNA sequences of visfatin were 5′‐UAAGGAAGGUGAAAUAUGAUU and 5′‐PUCAUAUUUCACCUUCCUUAUU, respectively. Scramble siRNAs of JNK and visfatin (Dharmacon) were used as negative control groups. HCAECs were transfected with siRNA oligonucleotides, using the Effectene transfection reagent (Qiagen).

### 2.9 Enzyme‐linked immunosorbent assay (ELISA) for AngII

The concentration of AngII was measured by a quantitative sandwich enzyme immunoassay (Phoenix Pharmaceutical, INC), with an anti‐AngII antibody (Santa Cruz Biotechnology).^29^ Conditioned medium from HCAECs subjected to HBO and those from control were collected for AngII measurement. The lower limit of detection of AngII was 0.5‐1.5 pg/mL. Both the inter‐assay and the intra‐assay coefficient of variance were <10%.

### 2.10 Electrophoretic mobility shift assay

Nuclear protein concentrations from cells were determined using Bio‐Rad protein assay. Consensus and control oligonucleotides (Santa Cruz Biotechnology Inc) were labelled by polynucleotide kinase incorporation of [γ‐^32^P]. After radiolabeling of oligonucleotides, the nuclear extracts (4 µg of protein in 2 µL of nuclear extract) were mixed with 20 pmol of [γ‐^32^P]‐labelled consensus or mutant oligonucleotide in a total volume of 20 µL. The samples were resolved on a 4% polyacrylamide gel. Gels were dried and imaged by autoradiography. Control experiments were performed with mutant oligonucleotides or cold oligonucleotides to compete with labelled sequences. HIF‐1α for EMSA was purchased from Santa Cruz Biotechnology Inc

### 2.11 Promoter activity assay

The visfatin gene was amplified with the forward primer, CCACCGACTCGTACAAG, and the reverse primer, GTG AGCCAGTAGCACTC. The amplified product was digested with the MluI and BglII restriction enzymes and ligated into the pGL3‐basic luciferase plasmid vector (Promega). Site‐specific mutations were confirmed by DNA sequencing. Plasmids were transfected into HCAECs using a low pressure‐accelerated gene gun (Bioware Technologies). Test (2 µg) and control plasmids (pGL4‐Renilla luciferase; 0.02 µg) were cotransfected with gene gun and replaced by normal culture medium. Following HBO treatment, cell extracts were prepared using the Dual Luciferase Reporter Assay System (Promega) and measured for dual luciferase activity with a luminometer (Turner Designs).

### 2.12 Migration assay

The migration activity of HCAECs was determined by using the method described as follows: HCAECs (5 × 10^4^ cells) were seeded in each 100 mm plates for overnight. After cell have completely attached, the cells were scraped with a line in the middle of the plate by a yellow tip. The width of the line gap is around 250‐280 µm. After scratching the cells, the medium was changed with fresh medium. After incubation at 37°C for 2 hours with or without HBO, each culture was photographed at a magnification of 100× with a microscope video system. We measured the widths of three places each gap and average of them.

### 2.13 Capillary‐like network formation assay

Capillary‐like network formation was performed in an in vitro culture system. Matrigel 250 µL (BD Biosciences) was coated onto a 24‐well culture plate to solidify at 37°C for 1 hour. HCAECs on a Matrigel matrix were exposed to 2.5 ATA of oxygen in a hyperbaric chamber for 2 hours at 37°C. After HBO treatment, cells were placed in a humidified incubator for 16 hours with an atmosphere of 5% CO_2_. Capillary‐like network formation was observed with a phase‐contrast microscope (Nikon).

### 2.14 Glucose uptake in cultured HCAECs

HCAECs were seeded on ViewPlate (Packard Instrument Co.) for 60 minutes at a density of 5 × 10^3^ cells/well in serum‐free medium overnight. Visfatin siRNA, SP600125, losartan or AngII were added to the medium. For glucose uptake, 0.1 mmol/L 2‐deoxy‐D‐glucose and 3.33 nCi/mL 2‐[1,2‐^3^H]‐deoxy‐D‐glucose (PerkinElmer Life) were added to HCAECs. Cells were washed with phosphate‐buffered saline twice. Non‐specific uptake was performed in the presence of 10 µmol/L cytochalasin B and subtracted from the measured values. MicroScint‐20 (50 µL) was added, and the plate was read with Top Count (Packard Instrument Co.). Radioactivity was counted and normalized to the protein amount as measured with a protein assay kit.

### 2.15 Statistical analysis

Data were expressed as mean ± SD. Statistical significance was performed with analysis of variance (GraphPad Software Inc). The Tukey‐Kramer comparison test was used for pairwise comparisons between multiple groups after the ANOVA. A value of *P* < .05 was considered to denote statistical significance.

## 3 RESULTS

### 3.1 Hypoxia increases visfatin expression in HCAECs

**Figure 1:**
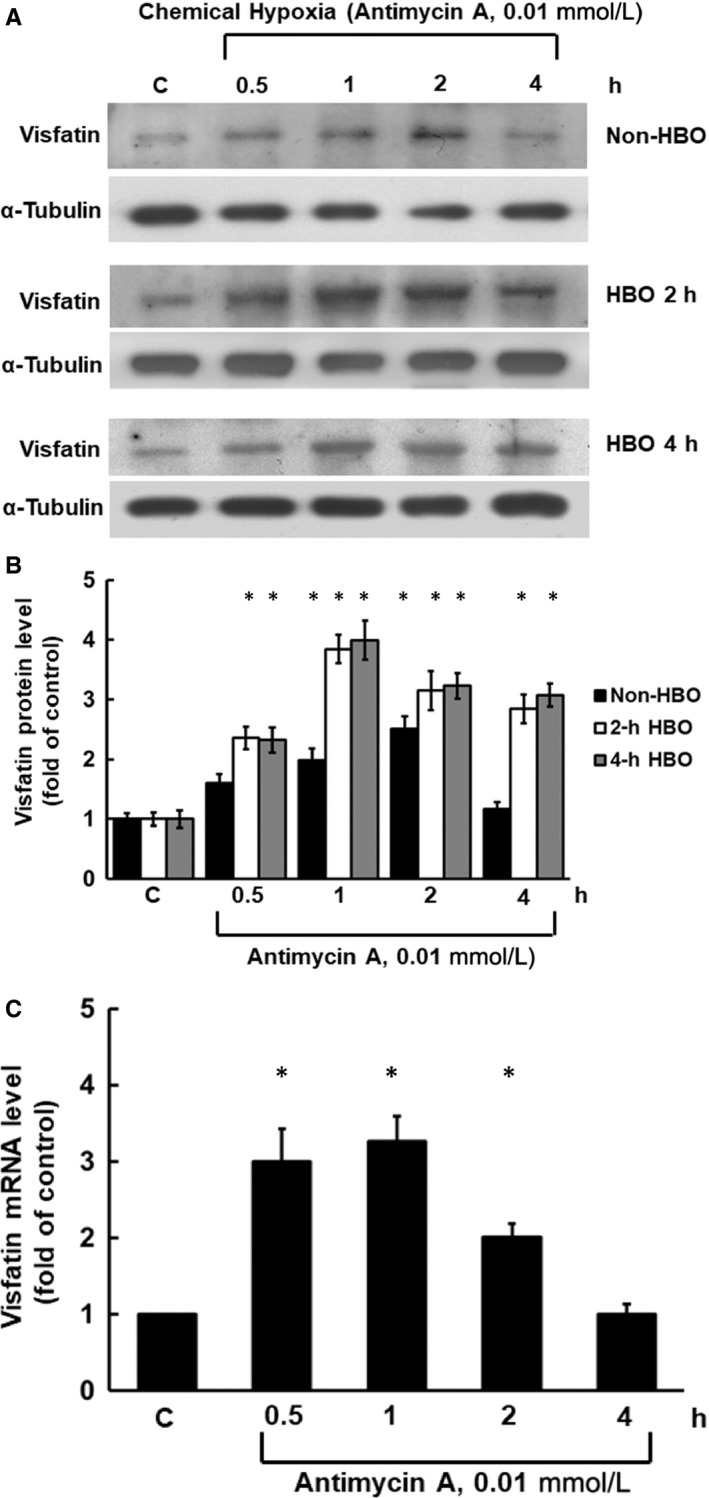
Hypoxia and HBO increase visfatin protein and mRNA expression in a time‐dependent manner. A, Representative Western blot for visfatin in HCAECs treated with chemical hypoxia (antimycin A, 0.01 mmol/L) and HBO (2.5 ATA) for 2‐4 h. B, Quantitative analysis of visfatin protein levels (n = 4). *P < .01 vs control. C, Quantitative analysis of visfatin mRNA levels. The values from treated HCAECs have been normalized to matched tubulin measurements and then expressed as a ratio of normalized values to mRNA in the control cells (n = 4). *P < .05 vs control. ATA, atmosphere absolute; HBO, hyperbaric oxygen; HCAEC, human coronary artery endothelial cell

HCAECs were exposed to chemical hypoxia for different periods of time (0.5, 1, 2 and 4 hours; Figure 1, n = 4). Visfatin protein expression increased after 0.5 hour of hypoxia (1.60 ± 0.5‐fold compared to control) and reached its maximal level after 2 hours (2.50 ± 0.22‐fold; Figure 1A,B). Under hypoxia, visfatin mRNA expression increased after 0.5 hour (3.00 ± 0.44‐fold) and reached its maximal level after 1 hour (3.26 ± 0.33‐fold; Figure 1C).

### 3.2 HBO promotes earlier and greater vistafin expression in hypoxic HCACEs

To investigate the effect of HBO on visfatin expression in hypoxic HCAECs, HBO treatment was applied for 2‐4 hours (Figure 1). Visfatin expression increased significantly under hypoxia for 1‐4 hours. After 2‐4 hours of HBO treatment, visfatin protein expression further increased in HCAECs exposed to 0.5 hours of hypoxia (2.36 ± 0.19 and 2.32 ± 0.21‐fold, respectively) and reached its maximal level after 1 hour of hypoxia (3.84 ± 0.24 and 3.99 ± 0.33‐fold; Figure 1A,B). After 2‐4 hours of HBO treatment, visfatin mRNA expression in HCAECs reached its maximal level after 0.5 hour of hypoxia (3.64 ± 0.27 and 3.73 ± 0.4‐fold; Figure 1C).

### 3.3 Increased visfatin protein expression in hypoxic HCAECs is mediated by the JNK pathway

**Figure 2:**
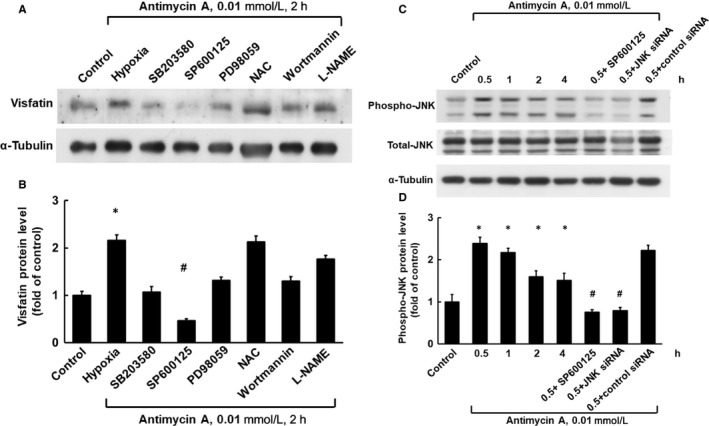
Hypoxia‐induced visfatin protein expression in HCAECs is primarily mediated via the JNK pathway. A, Representative Western blots for visfatin protein levels in HCAECs subjected to chemical hypoxia for 2 h in the absence or presence of inhibitors. B, Quantitative analysis of visfatin protein levels (n = 4). *P < .05 vs control. ^#^ *P* < .05 vs hypoxia. C, Representative Western blots for phospho‐JNK and total JNK protein levels in HCAECs subjected to chemical hypoxia for 0.5‐4 h in the absence or presence of inhibitors or siRNA. D, Quantitative analysis of phospho‐JNK protein levels (n = 4). *P < .05 vs control. ^#^ *P* < .05 vs hypoxia. JNK, c‐Jun N‐terminal kinase; siRNA, small interfering RNA

As shown in Figure 2A,B (n = 4), hypoxia‐induced visfatin protein expression was significantly reduced after the addition of SP600125 30 minutes before hypoxia and was also partially inhibited by SB203580 (P38 MAPK inhibitor), PD98059 (ERK inhibitor) and wortmannin (PI‐3K inhibitor). NAC and L‐NAME did not inhibit hypoxia‐induced visfatin expression. The finding implies that the JNK pathway is the main mediator of visfatin protein expression in hypoxic HCAECs. We then investigated phosphorylation of the JNK pathway during visfatin protein expression induced by hypoxia (Figure 2C,D, n = 4). Hypoxic HCAECs significantly increased the phosphorylation of JNK. SP600125 and JNK siRNA significantly attenuated the increased phosphorylation of JNK induced by hypoxia.

### 3.4 Hypoxia‐ and HBO‐induced visfatin protein expression in HCAECs is mediated by AngII

**Figure 3:**
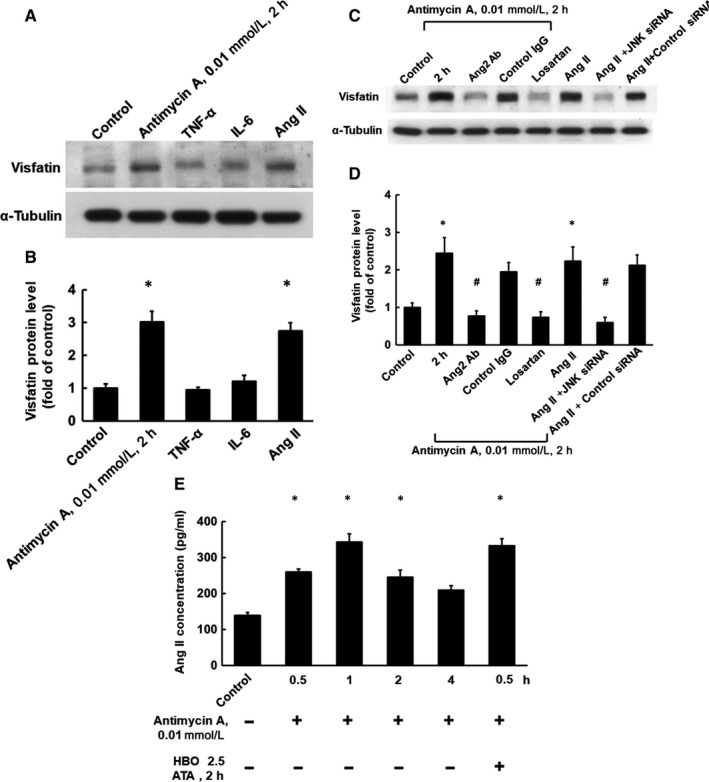
AngII increases visfatin expression. A, Representative Western blot for visfatin in HCAECs treated with hypoxia and different cytokines. B, Quantitative analysis of visfatin protein levels (n = 4). *P < .01 vs control. C, Representative Western blots for visfatin protein levels in HCAECs subjected to chemical hypoxia for 2 h or treated with AngII in the presence or absence of AngII antibody, ARB (losartan) or JNK siRNA. D, Quantitative analysis of visfatin protein levels (n = 4). *P < .05 vs control. ^#^ *P* < .05 vs hypoxia for 2 h. E, HBO enhanced AngII secretion from hypoxic HCAECs. Secretion of AngII was measured by ELISA (n = 4). *P < .01 vs control. AngII, angiotensin II; ARB, AngII receptor blocker; ELISA, enzyme‐linked immunosorbent assay

As shown in Figure 3A,B (n = 4), both hypoxia and exogenously added AngII significantly increased visfatin protein expression in HCAECs. However, exogenously added TNF‐*α* or IL‐6 did not increase visfatin protein expression. Hypoxia‐induced visfatin protein expression could be effectively inhibited by AngII antibody or losartan. Furthermore, exogenously added AngII‐induced visfatin protein expression was blocked by JNK siRNA (Figure 3C,D, n = 4). We also found that hypoxia increased AngII secretion after 0.5 hour (259.3 ± 8.3 mmol/L) and reached its maximal level after 1 hour (343.3 ± 22.3 nmol/L). In addition, HBO treatment for 2 hour resulted in earlier and greater AngII secretion after 0.5 hour of hypoxia (323.3 ± 20.2 nmol/L; Figure 3E). These data indicate that AngII mediates the induction of visfatin expression by HBO in hypoxic HCAECs.

### 3.5 HBO increases HIF1α‐binding to the visfatin promoter and visfatin transcription in hypoxic HCAECs

**Figure 4:**
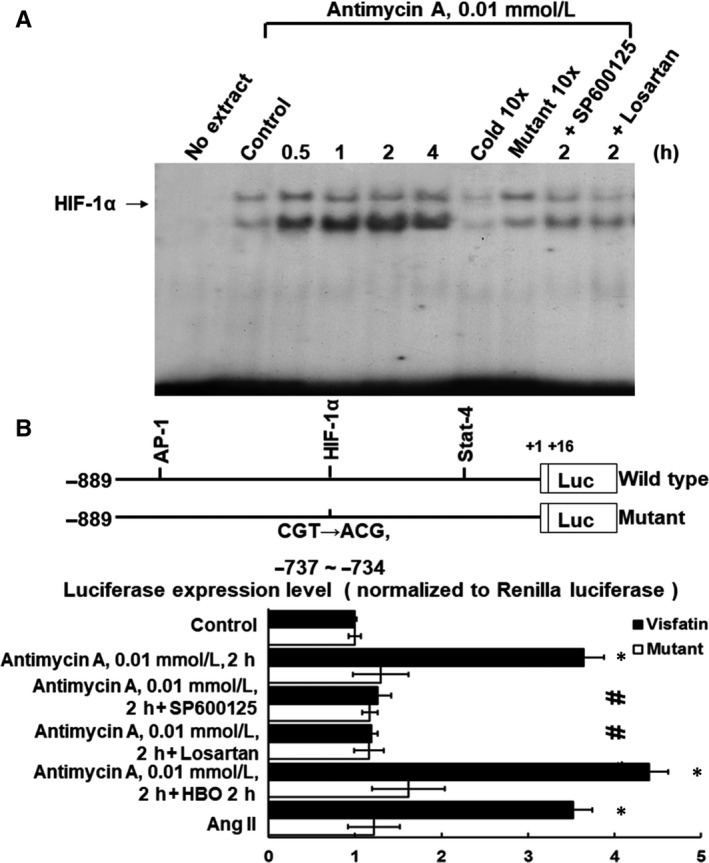
Hypoxia increases binding of HIF‐1α and transcriptional activity in the visfatin promoter. A, Representative EMSA showing protein binding to the HIF‐1‐α oligonucleotide in nuclear extracts of hypoxic HCAECs, in the presence or absence of the JNK inhibitor SP600125 or losartan (n = 4). Cold oligonucleotide refers to unlabelled HIF‐1α. B, Constructs of the visfatin promoter gene. Mutant visfatin promoter designates mutation of HIF‐1α binding sites in the visfatin promoter as indicated. C, Quantitative analysis of visfatin promoter activity. Luciferase activity was measured in cell lysates and normalized with renilla activity (n = 4). *P < .05 vs control. ^#^ *P* < .05 vs hypoxia for 2 h. HIF‐1α, hypoxia‐inducible factor‐1α; EMSA, electrophoretic mobility shift assay

Hypoxia significantly increased the DNA‐protein binding activity of HIF‐1α to the visfatin promoter (Figure 4A, n = 4). Addition of SP600125 or losartan before hypoxia abolished the DNA‐protein binding activity induced by hypoxia. To study whether the visfatin expression induced by HBO is regulated at the transcriptional level in hypoxic HCAECs, we cloned the promoter of human visfatin (−889 ~ +16) and constructed a luciferase reporter plasmid (pGL3‐Luc). The visfatin promoter construct contains HIF‐1α binding sites. Hypoxia, hypoxia with HBO and exogenously added AngII significantly induced visfatin promotor activity. Mutation of the HIF‐1α binding sites abolished the increased promotor activity induced by hypoxia or hypoxia with HBO. Moreover, addition of SP600125 and losartan caused an inhibition of transcription. These results suggest that the HIF‐1α binding site in the visfatin promoter is essential for Hypoxia‐ and HBO‐induced activation of visfatin transcription and that HBO and hypoxia regulate the visfatin promoter via HIF‐1α and the JNK pathway.

### 3.6 HBO with hypoxia increases visfatin expression in the nucleus, capillary‐like network formation and cell migration of HCAECs

**Figure 5:**
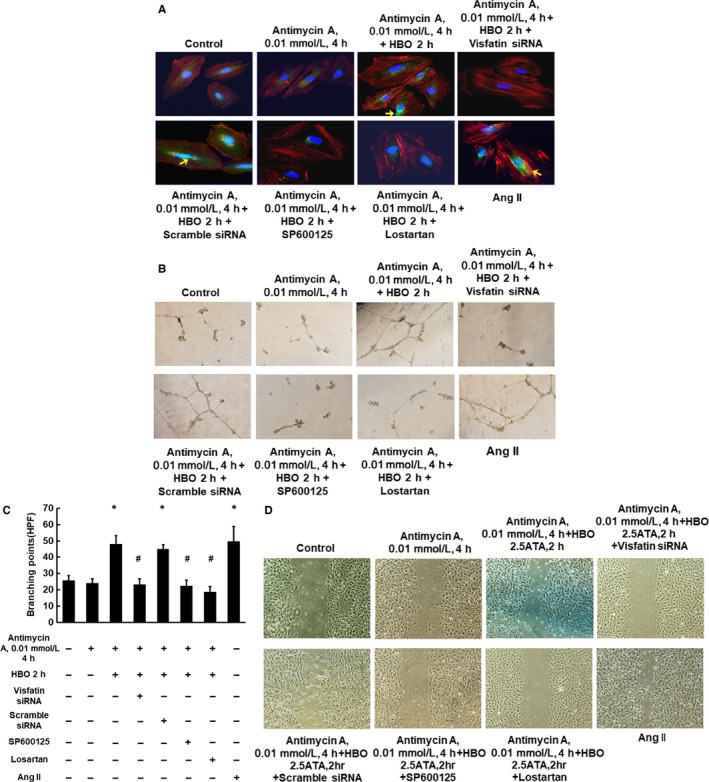
Effect of HBO with hypoxia on vistafin expression, capillary‐like network formation and cell migration of HCAECs. A, Confocal microscopy demonstrated the presence of visfatin (green) in the nuclei (blue) of HCAECs under hypoxia with HBO and in the presence of AngII. The effect of HBO on hypoxic HCAECs was inhibited by visfatin siRNA, SP600125 and losartan. B and C, Increased HCAEC tube formation under hypoxia with HBO and in the presence of AngII. HBO‐induced HCAEC tube formation was inhibited by visfatin siRNA, SP600125 and losartan (n = 4). *P < .05 vs control. ^#^ *P* < .05 vs hypoxia for 2 h. D, Cell migration assay showed increased migration of HCAECS under hypoxia with HBO for 2 h or exogenous addition of AngII. The cell migration effect of HBO on hypoxic HCAECs was inhibited by visfatin siRNA, SP600125 and losartan

As shown in Figure 5A (n = 4), confocal microscopy showed visfatin expression in the nucleus after hypoxia with HBO and exogenous addition of AngII. Visfatin siRNA and losartan inhibited nucleus expression of visfatin after hypoxia with HBO. Tube formation and numbers of branching points of HCAECs also increased after hypoxia with HBO and AngII (Figure 5B,C). Pre‐treatment with SP600125, losartan or visfatin siRNA significantly blocked hypoxia and HBO‐induced tube formation in HCAECs (Figure 5B,C, n = 4). Cell migration assay also showed increased migration of HCAECS under hypoxia with HBO or exogenous addition of AngII. Visfatin siRNA, SP600125 and losartan also inhibited cell migration after treatment with hypoxia and HBO (Figure 5D, n = 4).

### 3.7 Recombinant visfatin and HBO increase glucose uptake in hypoxic HCAECs

Hypoxia, hypoxia with HBO and AngII significantly increased glucose uptake at various periods of incubation when compared with untreated HCAECs (Figure S2, n = 4). This increase in glucose uptake was attenuated by addition of visfatin siRNA, SP600125 or losartan before hypoxia.

## 4 DISCUSSION

In this study, we demonstrated several significant findings about visfatin‐mediated angiogenesis in HCAECs under hypoxia and HBO treatment. Firstly, HBO further enhances visfatin expression in hypoxic HCAECs. Secondly, AngII, rather than IL‐6 or TNF‐α, acts as a pro‐inflammatory cytokine and cooperates with JNK pathway to mediate HBO‐induced visfatin expression in hypoxic HCAECs. Thirdly, AngII, the JNK pathway and HIF‐1α are involved in the signalling pathways mediating visfatin expression in response to HBO in hypoxic HCAECs. Fourthly, HBO increases tube formation and migration activity of hypoxic HCAECs to promote angiogenesis.

In our study, both the JNK pathway and HIF‐1α are responsible for hypoxia‐induced visfatin expression modulated by HBO in HCAECs. The vascular effects of visfatin include angiogenesis and endothelial regulation. However, different pathways of visfatin‐related angiogenesis were reported in previous studies.^20^, ^32^ In atherosclerotic plaques, visfatin stimulates MCP‐1, leading to angiogenesis through NF‐KB.^20^, ^32^ Chronic treatment with visfatin has been reported to induce angiogenesis in ECs both in vivo and in vivo, providing neovascularization of atherosclerotic plaques.^9^ Another mechanism by which visfatin has been shown to promote angiogenesis is through increasing NO production in human ECs, via Src, ERK1/2 and PI3K–Akt pathways.^13^ However, our study showed that L‐NAME did not effectively inhibit hypoxia‐induced visfatin expression, suggesting that NO was not involved in hypoxia‐induced visfatin expression in HCAECs in our study.

Pro‐inflammatory cytokines have been demonstrated to mediate visfatin‐induced angiogenesis. In human ECs, IL‐6 is up‐regulated by visfatin‐activated Janus kinase 2‐STAT3 and chemokine ligand 2 (CCL2).^7^ Both IL‐6 and CCL2 have been shown to be up‐regulated by visfatin via PI3K, ERK1/2 and p38 MAPK activation in human ECs.^35^ Our previous study also demonstrated that HBO‐induced visfatin expression in HCAECs is mediated by TNF‐*α* through the JNK pathway.^27^ We tested for HCAEC stimulation in response to three pro‐inflammatory cytokines and found that AngII, rather than IL‐6 or TNF‐*α*, acted in our model of HBO‐induced visfatin expression in hypoxic HCAECs. Different pro‐inflammatory cytokines may regulate HBO‐induced visfatin expression in HCAECs with or without hypoxia. Our explanation is that TNF‐*α,* angiopoietin‐1 (AP‐1) and the JNK pathway induce visfatin expression under HBO with normoxia.^27^ However, AngII, HIF‐1α and the JNK pathway increase visfatin expression under hypoxia with HBO. Furthermore, HBO enhances greater and earlier visfatin expression in hypoxic HCAECs.

Visfatin induces monocyte adhesion to vascular ECs, presumably because of the up‐regulation of intercellular adhesion molecule 1 and vascular cell adhesion molecule 1 expression via ROS‐dependent NF‐κB signalling.^33^, ^34^, ^35^, ^36^ Nicotinamide adenine dinucleotide phosphate (NADPH) oxidase is the major source of visfatin‐induced ROS generation in human ECs.^37^ Xia et al^35^ reported a mechanism for visfatin‐induced endothelial regulation in coronary artery ECs, through lysosome‐dependent lipid‐raft signalling and activation of NADPH oxidase, leading to ROS production. However, our study showed that NAC did not effectively inhibit visfatin expression under hypoxia, suggesting that ROS production was not related to visfatin expression in hypoxic HCAECs in our study.

Our study confirmed the stimulation of angiogenesis in hypoxic HCAECs after HBO treatment through HIF‐1α‐related visfatin expression. Our study also demonstrated that AngII secretion increased in hypoxic HCAECs and was modulated by HBO. SP600125 and ARB attenuated the HBO‐induced increase in visfatin expression. HIF‐1α is a principal transcriptional factor activated by AngII and is also considered one of the downstream targets of JNK. In this study, we demonstrated that HBO increased HIF‐1α binding activity. ARB and SP600125 inhibited the HIF‐1α binding activity induced by hypoxia, indicating that AngII and the JNK pathway mediate the transcriptional activity of HIF‐1α. Furthermore, ARB and SP600125 attenuated the HBO‐induced increase in visfatin promoter activity in hypoxic HCAECs. Our data also indicated that the HIF‐1α binding site in the visfatin promoter is essential for transcriptional regulation by HBO. Thus, HIF‐1α may regulate hypoxia‐activated genes and play a pivotal role in cardiac protection.^36^

HBO may induce expression of different genes via its unique signal pathway, depending on cell type.^24^, ^25^ The use of hyperbaric oxygen pressure makes HBO a useful tool for the treatment of different diseases. In this study, HBO induced tube formation of hypoxic HCAECs, an essential part of angiogenesis, indicating that HBO may be beneficial for patients with refractory ischaemic cardiovascular diseases. In the present study, we found that HBO treatment stimulated migration, proliferation and angiogenesis in HCAECs. Visfatin siRNA attenuated the migration and proliferation of HCAECs induced by HBO. We found no significant cytotoxicity in response to hypoxia in HCAECs as compared to control cells (Figure S3). Visfatin has been demonstrated to improve insulin sensitivity.^3^ In this study, we demonstrated that HBO increases glucose uptake in hypoxic HCAECs, as previous reported effect of visfatin. Visfatin siRNA attenuated this HBO‐induced glucose uptake. This finding indicates that visfatin mediates glucose uptake by HBO in hypoxic HCAECs (Figure S2). Increased glucose uptake in response to HBO may improve energy metabolism in HCAECs.

In conclusion, our study reported for the first time that both hypoxic HCAECs and exogenously added AngII increase visfatin expression. HBO enhances visfatin expression and AngII secretion and increases migration and tube formation of hypoxic HCAECs. The HBO‐induced visfatin expression is mediated by AngII, mainly via the JNK pathway.

## CONFLICT OF INTEREST

The authors confirm that there are no conflicts of interest.

## AUTHOR CONTRIBUTIONS

CC, BW and KS designed the study. CC, BW, YY and KS conducted acquisition of all the basic data. BW and YY analysed and did statistics for the data. CC, BW and KS supervised manuscript preparation and edited the manuscript.
