# Supplementary figures and images for: Hyperbaric oxygen activates visfatin expression and angiogenesis via angiotensin II and JNK pathway in hypoxic human coronary artery endothelial cells
Source: J Cell Mol Med. 2020 Jan 19;24(4):2434–43. doi: 10.1111/jcmm.14926 (PMC7028865; doi:10.1111/jcmm.14926)

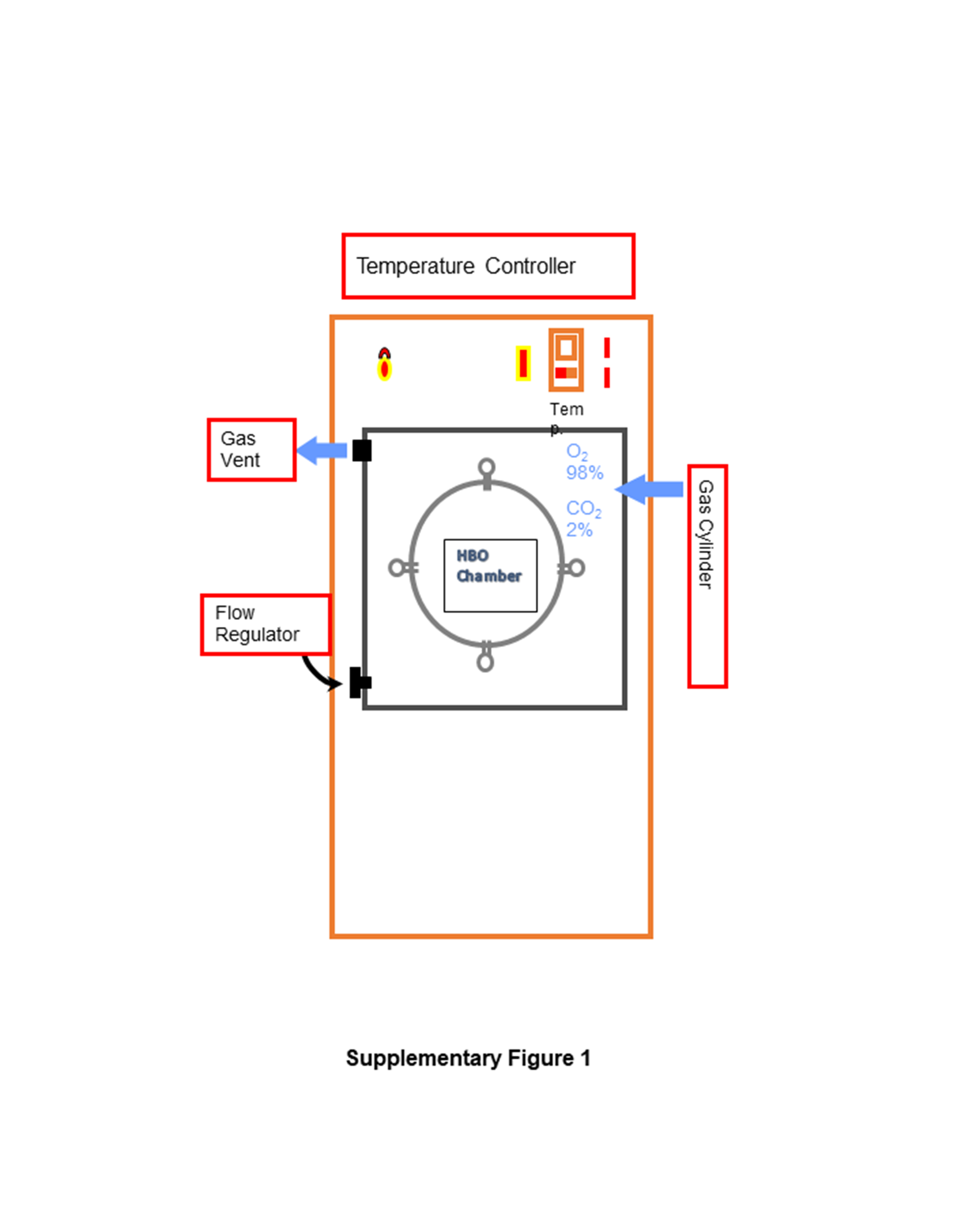

Supplement: Supplementary file 1 [file JCMM-24-2434-s001.TIF]

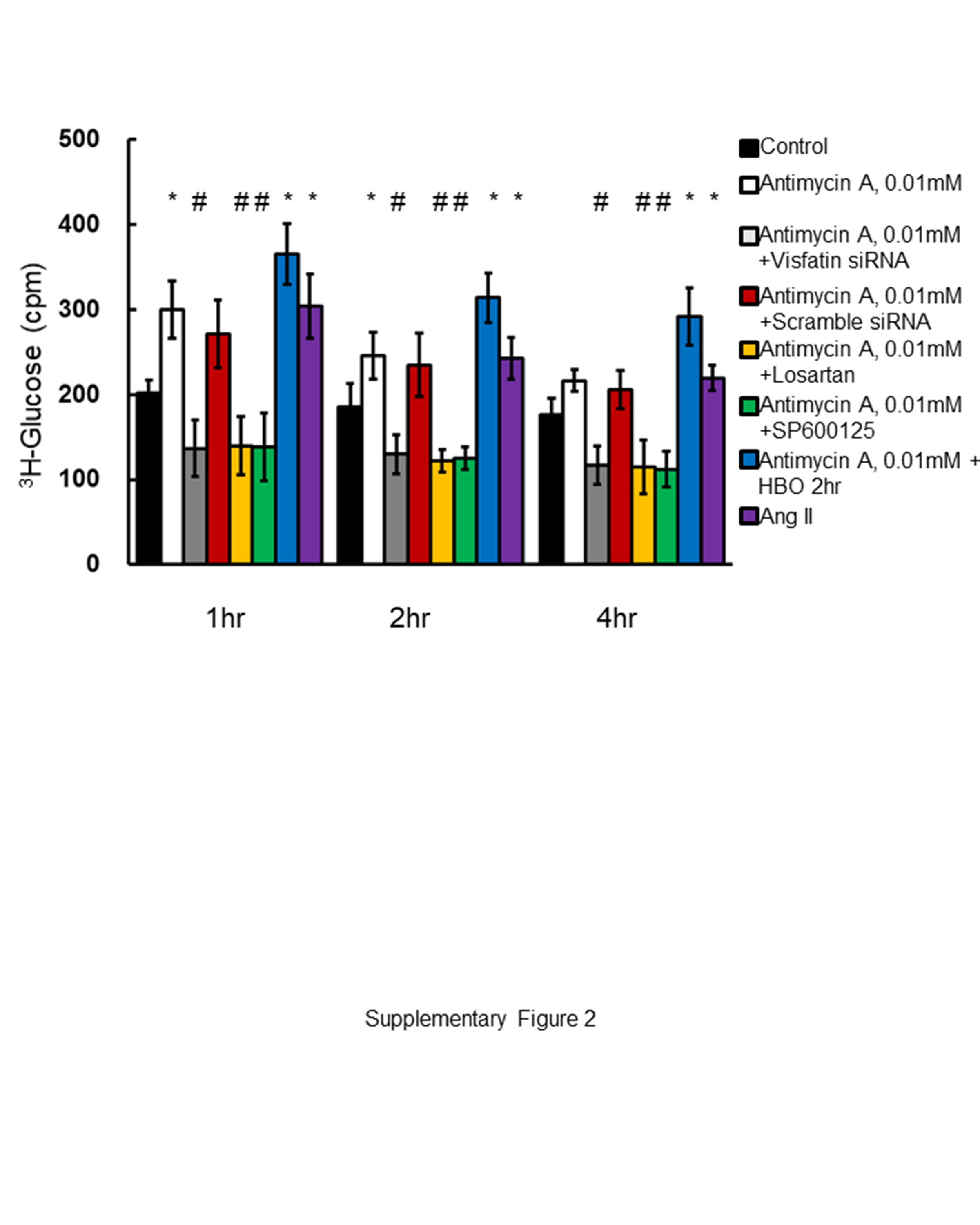

Supplement: Supplementary file 2 [file JCMM-24-2434-s002.TIF]

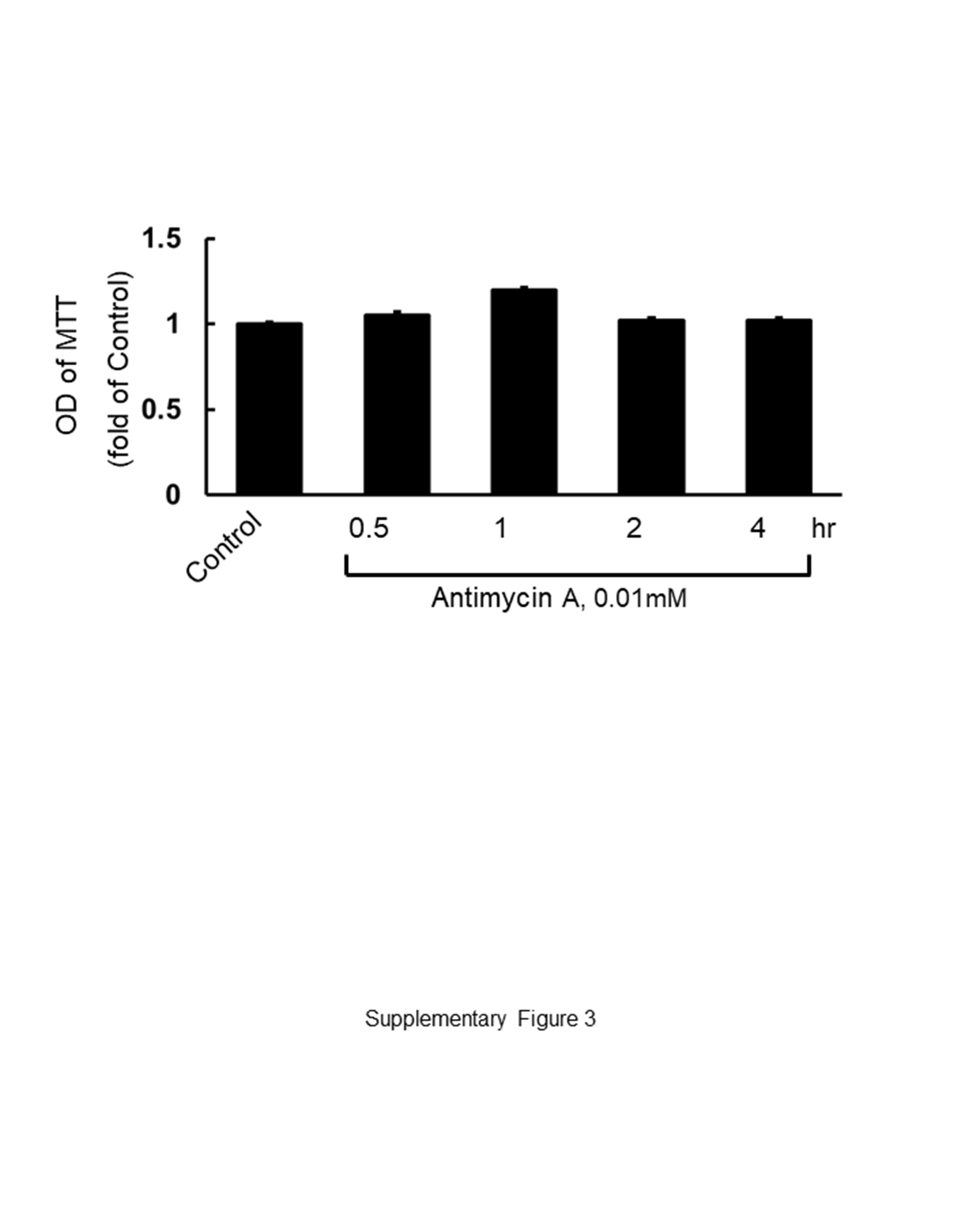

Supplement: Supplementary file 3 [file JCMM-24-2434-s003.TIF]
