# Supplementary material for: Hyperbaric oxygen activates visfatin expression and angiogenesis via angiotensin II and JNK pathway in hypoxic human coronary artery endothelial cells
Source: J Cell Mol Med. 2020 Jan 19;24(4):2434–43. doi: 10.1111/jcmm.14926 (PMC7028865; doi:10.1111/jcmm.14926)
Supplement: Supplementary file 4 [file JCMM-24-2434-s004.docx]

**Supplementary figure legends**

**Supplementary Figure 1:** Schematic diagram of the hyperbaric chamber in the incubator.

**Supplementary Figure 2:** Effect of HBO on glucose uptake in HCAECs.
Glucose uptake was measured in HCAECs after exposure to hypoxia, hypoxia with HBO, and AngII for 1–4 h. Hypoxia-induced glucose uptake in HCAECs was inhibited by visfatin siRNA, SP600125, and losartan. *P < 0.05 vs. control; ^#^P < 0.05 vs. hypoxia (n = 4).

**Supplementary Figure 3:** Quantitative analysis of cell viability under chemical hypoxia for 0.5–4 h, measured by MTT assay. There was no difference between hypoxic and control cells (n = 4). MTT = (3-(4,5-Dimethylthiazol-2-yl)-2,5- diphenyltetrazolium bromide.
